# Role of postoperative radiotherapy in reducing ipsilateral recurrence in DCIS: an observational study of 1048 cases

**DOI:** 10.1186/s13014-018-0964-7

**Published:** 2018-02-09

**Authors:** Stefanie Corradini, Montserrat Pazos, Stephan Schönecker, Daniel Reitz, Maximilian Niyazi, Ute Ganswindt, Simone Schrodi, Michael Braun, Martin Pölcher, Sven Mahner, Nadia Harbeck, Jutta Engel, Claus Belka

**Affiliations:** 1 Department of Radiation Oncology, University Hospital, LMU Munich, Marchioninistraße 15, 81377 Munich, Germany; 2 Department of Therapeutic Radiology and Oncology, Innsbruck Medical University, Innsbruck, Austria; 3 Munich Cancer Registry (MCR) of the Munich Tumour Centre (TZM) at the Institute of Medical Information Processing, Biometry and Epidemiology (IBE), University Hospital, LMU Munich, Marchioninistr. 15, 81377 Munich, Germany; 4 Red Cross Breast Centre, Taxisstr. 3, 80637 Munich, Germany; 5 Department of Gynecology and Obstetrics, Breast Centre, University Hospital, LMU Munich, Marchioninistr. 15, 81377 Munich, Germany; 6 Comprehensive Cancer Center (CCC-LMU), University Hospital, LMU Munich, Marchioninistr. 15, 81377 Munich, Germany

**Keywords:** Ductal carcinoma in situ, Breast conserving surgery, Radiotherapy, Local control, In-breast recurrence, Survival, Outcome

## Abstract

**Background:**

The objective of the present study was to evaluate the effectiveness of postoperative radiotherapy after breast conserving surgery (BCS) in DCIS in a large patient population treated in clinical practice.

**Methods:**

Data were provided by the population-based Munich Cancer Registry. Between 1998 and 2014, 1048 female patients with diagnosis of DCIS and treated at two Breast Care Centres were included in this observational study. The effectiveness of postoperative radiotherapy and variables predicting the use of radiotherapy were retrospectively analysed.

**Results:**

After adjusting for age, tumour characteristics and therapies, Cox regression analysis for local recurrence-free survival identified RT as an independent predictor for improved local control (HR: 0.579; 95%CI: 0.384–0.872, *p* = 0.008). Ten-year cumulative incidence of in-breast recurrences was 20.0% following BCS, compared to 13.6% in patients receiving postoperative radiotherapy (*p* = 0.012). As an estimate for disease-specific survival, 10-year relative survival was 105.4% for patients receiving postoperative radiotherapy and 101.6% without radiotherapy. On multivariate analysis, postoperative radiotherapy was not associated with improved overall survival (HR 0.526; 95%CI: 0.263–1.052, *p* = 0.069). Over time, a significant increase of RT was registered: while 1998 only 42.9% of patients received postoperative radiotherapy, the proportion rose to 91.2% in 2014. Women aged < 50 years (OR: 2.559, 95%CI: 1.416–4.625, *p* < 0.001) or with negative hormone receptor status (OR: 2.625, 95%CI: 1.458–4.728, *p* = 0.001) or receiving endocrine therapy (OR: 1.762, 95%CI: 1.060–2.927, *p* = 0.029) were more likely to receive postoperative radiotherapy after BCS.

**Conclusions:**

In conclusion, this study provides insights regarding the adoption and treatment pattern of postoperative RT following BCS for DCIS in a large cohort reflecting “real-life” clinical practice in this setting. Postoperative RT was found to be associated with a reduced risk of ipsilateral recurrence and no survival benefit compared to observation alone.

## Background

The optimal management of ductal carcinoma in situ (DCIS) of the breast is still controversial and to date, inconsistent and conflicting results have been reported across prospective and observational studies [1]. As known from several randomised trials and a large EBCTCG meta-analysis [2–6] postoperative radiotherapy (RT) after breast conserving surgery is a very effective adjuvant local treatment modality. Radiotherapy approximately halves the risk of local recurrences [6] as compared to surgical resection alone, with similar effects in reducing ipsilateral in situ and invasive recurrences [4]. This effect was observed in all subgroups of patients and could not be translated into a survival benefit in randomised studies [6]. Therefore, over the past years a risk-benefit debate for or against postoperative radiotherapy has attracted wide public attention [1, 7].

DCIS of the breast represents a very heterogeneous disease, making a risk-group stratification challenging. Considerable efforts have been spent in the implementation of risk-adapted treatment concepts. One major objective was to identify a subset of “low-risk” patients with excellent prognosis, where a treatment de-escalation with omission of radiotherapy can be safely attempted, as recurrence rates are very low [8, 9]. On the other hand, considerations about a possible undertreatment of “high-risk” patients have led to treatment escalation strategies using boost irradiation to the tumour bed to further increase local control rates [10, 11].

The above-mentioned uncertainties result in a vast heterogeneity of criteria influencing treatment decision-making in real-world settings. Clinicians have to choose from a range of available risk stratification tools [12–14] and treatment options in order to select a suitable treatment strategy for each individual patient. Furthermore, shared decision-making in daily clinical practice is strongly influenced by a number of confounding factors, such as clinician and patient preferences and trade-offs concerning toxicity risks or comorbidities [15–17]. The aim of the present study was to evaluate the effectiveness of postoperative radiotherapy in DCIS in a large patient population, outside of clinical trials. Moreover, the study aimed to identify possible factors related to the use of postoperative radiotherapy in daily clinical practice.

## Methods

### Data sources

Data were retrieved from the Munich Cancer Registry (MCR) [18]. The catchment area of the population-based clinical cancer registry to date encompasses 4.81 million inhabitants in southern Germany. Over the past decades it has been stepwise enlarged from 2.3 million inhabitants in 1998 to 3.84 million in 2002, and nowadays includes all regions of Upper Bavaria. The MCR systematically retrieves cancer notifications from 73 MCR-affiliated hospitals or other notifying institutions. Patient’s demographics, cancer diagnosis, disease and treatment characteristics and follow-up are reported following official documentation guidelines for cancer registries [19, 20]. Follow-up was conducted following the national German “S3-guideline” for breast cancer [21], with regularly clinical and mammographic follow-ups. All follow-up data were provided as cancer notifications from MCR-affiliated hospitals or other notifying institutions on a regular basis and collected prospectively at the MCR. Moreover, all pathology laboratories within the catchment area of the MCR are required to submit their pathology reports to the cancer registry. Thus, the total number of pathologically diagnosed recurrences within the catchment area was systematically obtained. Survival information is maintained systematically from death certificates of 23 health offices within the catchment area of the MCR.

### Study population

For the present study, patients treated at the Red Cross Breast Centre and the LMU Breast Centre in Munich (Germany) between 1998 and 2014 were analysed. Of all breast malignancy records, male patients (*n* = 63), cases with histology of lymphoma (*n* = 10), sarcoma (*n* = 58) or invasive carcinoma (*n* = 13,064), or with unknown date of initial diagnosis (e.g. tumors from death certificate information only [DCO]) (*n* = 58), as well as cases with a previous diagnosis of cancer or simultaneous cancer diagnoses (*n* = 3200) were excluded from the analysis. Of the remaining cases, patients who underwent mastectomy (*n* = 296), did not undergo any surgery (*n* = 8), or cases where surgery information or pathologic tumour stage was incomplete or missing (*n =* 58) were not included in the present study. The final study cohort encompassed 1048 patients, who underwent breast conserving surgery for ductal carcinoma in situ over a 17-year period at two Breast Centres. Follow-up was completed on October 5th 2016.

### Statistical analyses and endpoints

Statistical analyses were conducted using IBM SPSS Statistics 24.0 and R environment for statistical computing and visualization (version. 3.4.0). Patient and treatment characteristics were analysed using descriptive statistics and analysed using the Chi Square test. The percentages of the presented subcategories were related to the sum of available data of each variable and did not consider missing values. Primary endpoints were the impact of postoperative radiotherapy on local control and overall survival (OS). In-breast recurrence (IBR) was defined as invasive or in-situ recurrence in the ipsilateral breast. To account for competing risks, cumulative incidence analysis (CI) was used to calculate time to IBR and differences were assessed by Gray’s Test for Equality of Cumulative Incidence Functions. OS was estimated using the Kaplan-Meier method and compared using the log-rank test. Relative survival was calculated by the ratio of the overall survival rate to the expected survival rate and 95% confidence intervals (95%-CI) were used for assessing significance. To account for competing risks, Cox proportional hazards models were used to identify independent prognostic factors related to local recurrence free survival (LRFS) and OS. Furthermore, factors influencing the use of postoperative radiotherapy in DCIS were determined by using a multivariate logistic regression analysis. The significance level in all analyses was set at 5%.

## Results

**Fig. 1:**
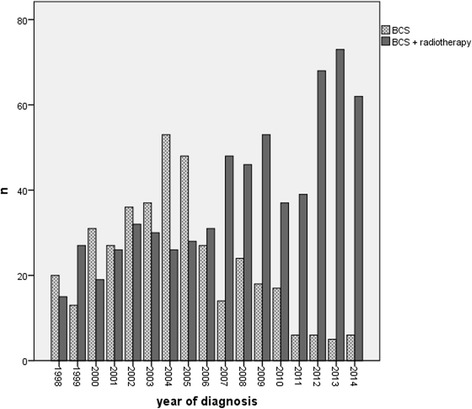
The use of postoperative radiotherapy over time

Patient and treatment characteristics of 1048 patients diagnosed with ductal carcinoma in situ between 1998 and 2014 are summarized in Table 1. Median follow-up was 88.0 months; 69.0 months for the radiotherapy group and 123.0 months for patients receiving BCS alone. Overall, 388 patients underwent breast conserving surgery alone, while 660 patients were treated with additional postoperative radiotherapy of the ipsilateral breast. Age at diagnosis was significantly different between the groups (*p* < 0.001). Patients in the BCS group were significantly older, with a median age of 58.8 years (range: 30–90), as compared to a median age of 57.3 years (range: 28–89) in patients undergoing BCS and postoperative radiotherapy. While tumour side was balanced between the two cohorts, there were a number of significant differences between patients undergoing BCS and patients receiving additional postoperative radiotherapy. High tumour grade (G3) was present in 29.3% of the surgery only group. The proportion was significantly higher in patients undergoing radiotherapy, presenting with 49.9% grade 3 tumours (*p* < 0.01). Similarly, negative hormone receptor status was more frequent in the radiotherapy group, as compared to patients not receiving radiotherapy (17.3% vs 10.4%, *p* = 0.012). Nodal involvement (pN1) was present in 2 irradiated patients (0.3%), while there was no case in the BCS group. Furthermore, patients in the radiotherapy cohort underwent surgical axillary intervention more frequently (sentinel lymph node biopsy or axillary dissection) as compared to the BCS cohort (18.2% vs. 10.6%, *p* = 0.016). Regarding other treatment modalities, irradiated patients received endocrine therapy in 18%. In contrast, in patients receiving no radiotherapy, adjuvant endocrine therapy was administered in 8.5% (*p* < 0.001). A significant increase in the use of postoperative radiotherapy was documented over time (see Fig. 1). In 1998, only 42.9% of patients received postoperative radiotherapy following BCS, whereas the proportion increased to 91.2% by 2014. The standard radiotherapy regimen at the Department of Radiation Oncology of LMU University was whole-breast irradiation (50.4 Gy in 28 fractions or 50 Gy in 25 fractions) followed by a boost of 10–16 Gy to the tumour bed in high-risk cases with close resection margins.

**Table 1:** Cohort characteristics of the different treatment groups. BCS: breast conserving surgery

	BCS		BCS + Radiotherapy		
	388 patients		660 patients		
	*n*	(%)^a^	*n*	(%)^a^	*p*-value
Age at diagnosis (years)					
< 50	96	(24.7)	150	(22.7)	< 0.001
50–69	224	(57.8)	448	(67.9)	
≥ 70	68	(17.5)	62	(9.4)	
median age (years)	58.8		57.3		
Tumor side					
Left	217	(56.5)	354	(53.6)	0.368
Right	167	(43.5)	306	(46.4)	
*unknown*	*[4]*	*[1.0]*			
Tumour stage					
pTis	388	(100)	660	(100)	
Tumour size					
< 25 mm	26	(74.3)	41	(66.1)	0.404
≥ 25 mm	9	(25.7)	21	(33.9)	
*unknown*	*[353]*	*[90.9]*	*[598]*	*[90.6]*	
Nodal status					
pN0	45	(11.6)	139	(21.1)	< 0.001
pN1	0	(0.0)	2	(0.3)	
pNx/unknown	343	(88.4)	519	(78.6)	
Grade					
G1	44	(29.9)	63	(13.3)	< 0.001
G2	60	(40.8)	175	(36.8)	
G3	43	(29.3)	237	(49.9)	
*unknown*	*[241]*	*[62.1]*	*[185]*	*[28.0]*	
Multifocality					
no	365	(94.1)	633	(95.9)	0.178
yes	23	(5.9)	27	(4.1)	
Resection margins					
R0	235	(97.1)	554	(96.5)	0.666
R1–2	7	(2.9)	20	(3.5)	
*Rx/unknown*	*[146]*	*[37.6]*	*[86]*	*[13.0]*	
Hormone Receptor					
positive	215	(89.6)	492	(82.7)	0.012
negative	25	(10.4)	103	(17.3)	
*unknown*	*[148]*	*[38.1]*	*[65]*	*[9.8]*	
Endocrine therapy					
no	355	(91.5)	542	(82.1)	< 0.001
yes	33	(8.5)	118	(17.9)	

^a^Percentages of the presented subcategories are related to the sum of each item with available data; missing values are not taken into account

**Fig. 2:**
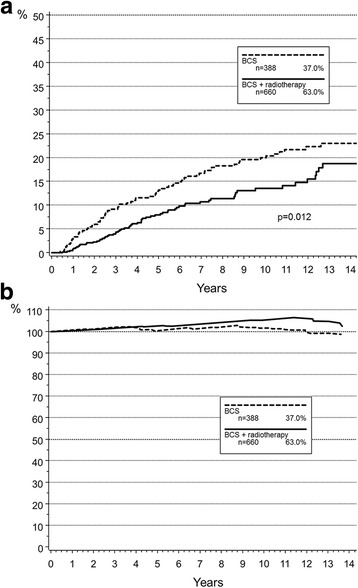
Cumulative incidence of in-breast recurrences (**a**) and relative survival (**b**)

During follow-up, 93 patients developed a DCIS recurrence and 50 patients an invasive ipsilateral recurrence. Median time to in-situ recurrence was 45 months (range: 5–149 months) and 69 months (range: 11–152 months) for invasive IBR. In-breast recurrence rates were significantly influenced by postoperative radiotherapy (Table 2). Cumulative incidence (CI) analysis showed a 10-year CI of IBR of 20.0% following BCS, compared to 13.6% in patients receiving postoperative radiotherapy (*p* = 0.012, Fig. 2a). Overall, 10-year CI of DCIS IBR was 11.2% as compared to 6.5% for invasive recurrences. Other variables, including tumour age, size and grade, multifocality, resection status, hormone receptor status or the use of adjuvant endocrine therapy did not significantly influence outcome on univariate analysis. In multivariate Cox regression analysis accounting for confounding factors, postoperative radiotherapy remained independently associated with improved local recurrence free survival (hazard ratio [HR]: 0.579; 95%CI: 0.384–0.872, *p* = 0.008). Results are summarized in Table 3.

**Table 2:** Cumulative incidence of in-breast recurrences (IBR) and Kaplan-Meier estimates of overall survival (OS)

	IBR			OS		
	5 y (%)	10 y (%)	*p*	5 y (%)	10 y (%)	*p*
Age at diagnosis (years)						
< 50	12.6	17.6	0.251	100.0	98.5	< 0.001
50–69	9.7	16.4		98.2	95.1	
≥ 70	7.0	13.8		88.1	74.6	
Tumour localisation						
Left	9.4	14.8	0.134	97.0	93.0	0.830
Right	11.0	18.3		97.6	93.8	
Tumour size						
< 25 mm	12.6	17.5	0.274	96.7	94.9	0.225
≥ 25 mm	6.7	6.7		88.9	84.2	
Grade						
G1	12.0	22.4	0.212	93.3	93.3	0.444
G2	5.3	12.4		96.6	95.3	
G3	11.7	15.9		99.4	90.7	
Multifocality						
no	10.0	16.2	0.592	97.3	93.4	0.972
yes	11.1	19.4		97.6	93.1	
Resection margins						
R0	9.3	15.6	0.526	97.6	93.1	0.953
R1–2	14.8	20.2		96.2	96.2	
Hormone receptor status						
positive	8.6	16.2	0.719	97.2	94.3	0.501
negative	13.0	15.6		97.6	90.1	
Radiotherapy						
no	13.2	20.0	0.012	95.1	90.0	0.001
yes	8.0	13.6		99.1	96.7	
Endocrine therapy						
no	10.9	17.4	0.111	97.2	93.6	0.319
yes	5.5	10.7		97.6	92.3

**Table 3:** Multivariate Cox regression analysis for loco-regional recurrence-free survival (LRFS) and overall survival (OS) for DCIS

	LRFS			OS		
Variable	HR	95% CI	*p*	HR	95% CI	*p*
Age at diagnosis			0.089			< 0.001
< 50 years	1			1		
50–69 y	1.014	0.625–1.645		4.809	0.639–36.165	
≥ 70 y	0.423	0.180–0.994		34.505	4.594–259.164	
Radiotherapy			0.008			0.069
yes	0.579	0.384–0.872		0.526	0.263–1.052	
no	1			1		
Multifocality			0.818			0.790
yes	1			1		
no	1.125	0.412–3.077		0.822	0.194–3.482	
Resection margins			0.424			0.948
R0	1			1		
R1–2	1.449	0.583–3.602		1.049	0.246–4.474	
Endocrine Therapy			0.097			0.274
yes	1			1		
no	1.710	0.906–3.226		0.621	0.265–1.458	

Patients receiving postoperative radiotherapy had a higher 10-year OS (96.7%) compared to patients undergoing breast conserving surgery alone (90.0%) (*p* = 0.001). In order to estimate disease-specific survival rates, relative survival was calculated. Ten-year relative survival was 105.4% for patients receiving postoperative radiotherapy and 101.6% without radiotherapy (Fig. 2b). After adjusting for risk factors in the Cox proportional hazards regression model, postoperative radiotherapy was not associated with improved overall survival (HR 0.526; 95%CI: 0.263–1.052, *p* = 0.069). Other risk factors than age did not correlate with improved OS (Table 3).

### Factors associated with the use of postoperative radiotherapy

A multivariate logistic regression analysis was performed to identify factors associated with the administration of postoperative radiotherapy in our collective (Table 4). Variables associated with the use of postoperative radiotherapy included young age at diagnosis, negative hormone receptor status, and administration of adjuvant endocrine therapy. Women aged < 50 years were more likely to receive postoperative radiotherapy after BCS (OR 2.559, 95%CI 1.416–4.625, *p* < 0.001). Similarly, women presenting with negative hormone receptor status had higher odds for being irradiated following BCS (OR 2. 625, 95%CI 1. 458–4.728, *p* = 0.001). Furthermore, postoperative radiotherapy was more likely in patients receiving adjuvant endocrine therapy (OR 1. 762, 95%CI 1.060–2.927, *p* = 0.029).

**Table 4:** Adjusted Odd Ratios (OR) and 95% Confidence Intervals (CI) from the multiple logistic regression analysis for postoperative radiotherapy compared to omission of radiotherapy

Variable	OR	95% CI	*p*
Age at diagnosis			< 0.001
< 50 years	2.559	1.416–4.625	
50–69 y	2.761	1.701–4.481	
≥ 70 y	1		
Tumour localisation			0.403
Right	1		
Left	0.857	0.597–1.230	
Multifocality			0.778
no	1.117	0.519–2.406	
yes	1		
Resection margins			0.663
R0	1		
R1–2	1.290	0.410–4.061	
Hormone receptor status			0.001
Positive	1		
Negative	2.625	1.458–4.728	
Endocrine therapy			0.029
no	1		
yes	1.762	1.060–2.927	

## Discussion

The present observational study analysed data from a large patient population treated at two major German breast centers outside of clinical trials. Women treated with postoperative radiotherapy following BCS for DCIS of the breast were found to have a significantly lower rate of ipsilateral recurrences as compared to BCS alone (20.0% vs 13.6%). This difference is consistent with other observational and randomised studies [3, 22, 23]. For example, the population-based SEER data analysis of 1103 women diagnosed with DCIS by Warren et al. [22], found a risk of developing an ipsilateral recurrence of 11% for patients who received postoperative radiotherapy versus 15% for women undergoing BCS alone, at a mean follow-up of 91 months. Accordingly, the NSABP B-17 trial [3] documented a 15-year cumulative incidence of ipsilateral recurrences of 19% for the lumpectomy group compared to 8% for the additional postoperative radiotherapy group. Similar results were seen in the EORTC 10853 trial [23], where radiotherapy reduced the incidence of invasive local recurrences after DCIS from 13% to 8% (*p* < 0.01) after 10 years follow-up. The large EBCTCG meta-analysis [6] confirmed, that postoperative RT after breast conserving surgery approximately halves the risk of local recurrences as compared to surgical resection alone, with similar effects in reducing ipsilateral in situ and invasive recurrences.

It is well known, that postoperative RT reduces the risk of local recurrences for all subgroups, independent of age. Nevertheless, the EORTC trial [23] found that local recurrence rates were significantly influenced by age at diagnosis, and young age < 40 years was identified as an independent high-risk factor for ipsilateral recurrences on multivariate analysis. Furthermore, the beneficial effect of RT on local control seems to be modified by age, resulting in a larger risk reduction for elderly patients. In the Swedish SweDCIS trial [2], women younger than 52 years had a smaller absolute risk reduction as compared to older age groups. The EBCTCG meta-analysis [6] confirmed that the proportional reduction in the rate of ipsilateral breast recurrence achieved with radiotherapy was greater in older than in younger women. Lately, additional efforts have been undertaken to further increase local control rates for this “high-risk” group of young patients, for example by the addition of a boost to the tumour bed. A retrospective analysis of Moran et al. [10] showed that an additional RT boost had a significant benefit in decreasing in-breast recurrences in 4131 patients (HR 0.68, 95%CI: 0.50–0.91, *p* = 0.01). Data from current randomised trials are eagerly awaited [24, 25]. It remains unclear, how many patients received a boost in the present study population, as detailed information on radiation dose was not available from cancer registry data.

The effect of radiotherapy on survival in ductal carcinoma in situ of the breast remains unclear. As DCIS is a non-invasive precursor lesion, overall survival after DCIS is very good, resulting in breast cancer specific survival rates of over 95% after 20 years [2]. So far, no statistically significant effect on breast cancer-specific survival has been observed in randomised trials [6]. It has to be mentioned, that most randomised trials were not powered to find a difference in breast-cancer specific survival or overall survival [13]. Furthermore, early detection and treatment of in situ recurrences (salvage mastectomy) might reduce the difference in outcome and result in comparable survival rates. On the other hand, it is well known, that patients with a subsequent invasive local recurrence have a significantly increased mortality risk after such events. In the EORTC trial [23], mortality risk increased by a factor of five and breast cancer-specific mortality by a factor of 17 following an invasive local recurrence. In contrast to randomised evidence, Sagara et al. [13] recently reported the outcome of a large population-based study of 32,144 DCIS patients. In this study, the use of radiotherapy in high-risk patients (high nuclear grade, young age, large tumor size) was associated with a significant survival benefit when compared to observation alone (HR 0.73, 95%CI; 0.62–0.88, *p* = 0.003). After adjusting for risk factors in the Cox proportional hazards regression model in the present study, postoperative radiotherapy was not associated with improved overall survival (HR 0.526; 95%CI: 0.263–1.052, *p* = 0.069). In order to estimate disease-specific survival rates, relative survival was calculated by adjusting the observed overall survival to the expected survival rates from the general population in Germany. Each individual of the study population was matched by age, sex and calendar year with the expected mortality of national life tables. Estimates of 10-year relative survival were 105% for patients receiving postoperative radiotherapy. A relative survival higher than 100% indicates that non-cancer life expectancy was more favorable among patients within this study as compared to the general population. This “healthy user effect” is a known source of sampling bias in observational studies of early stage breast or prostate cancer [26–28]. Patients diagnosed with DCIS through a screening examination, generally tend to be healthier and have a longer life expectancy than the general population as they are concerned for their health, seek preventive services and might also partake in other healthy behaviors as well as follow medical advice.

The results have to be interpreted with caution, as any retrospective approach has inherent limitations: As known from other observational studies [15, 29], outcome in clinical effectiveness research is influenced by a number of unmeasured confounding factors and associations between treatments, and outcomes can result from confounding and have no causal correlation [30, 31]. We tried to control for all known confounders available in the registry, which may have influenced the clinical treatment decision-making process. Nonetheless, it is not possible to fully control for host-related factors including comorbidities, performance status, or clinician- and patient-related preferences. Therefore, an unavoidable limitation is the inherent potential for selection bias due to disease severity [32], considering that the severity of the disease (e.g. presence of high-risk factors) is a potential confounder influencing the indication for postoperative radiotherapy in DCIS. In fact, the postoperative radiotherapy group of the present study included significantly more patients presenting with high-risk features like high tumour grade or negative hormone receptor status. Efforts have been undertaken to account for these clinicopathological risk-score features in multivariate analysis. However, a proper risk-group stratification to categorize baseline risks for local recurrence was not possible, since molecular subtypes and histopathological parameters were not available for all patients from the database. A strength of our study might be, that - in contrast to the highly selected and homogeneous study populations of randomised trials - this observational study reflects a heterogeneous cohort of patients treated in real-world clinical practice.

In the present analysis, a significant increase over time (1998–2014) regarding the use of postoperative radiotherapy was observed. This change in the use of RT probably results from the publication of four randomised trials conducted in the early nineties [2–4, 23]. While mastectomy was the treatment of choice until the 1980s, these trials seem to have influenced treatment decisions in real-world clinical scenarios. Our analysis strongly confirms the change of treatment patterns: while in 1998 only 42.9% of patients received postoperative radiotherapy following BCS, this percentage has risen to 91.2% in 2014. Factors associated with the use of postoperative radiotherapy on multivariate logistic regression analysis were typical high-risk features, as young age at diagnosis or negative hormone receptor status. Similar results were reported by Subhedar et al. [33], who retrospectively analysed 2996 cases of DCIS undergoing BCS from 1978 to 2010 at a single institution. Despite a significant increase of RT use over time (*p* < 0.0001), recurrence rates have fallen over time, even in patients undergoing BCS alone. The authors postulate that advances in digital mammography and improvements in pathological assessment might contribute to the reduction in recurrence rates [33].

Unfortunately, there are no widely accepted guidelines helping to stratify patients in clinical practice. Further prospective studies will be needed to clarify the debated role of RT in DCIS and assist clinicians to tailor risk-adapted RT strategies for their DCIS patients. In clinical practice, thorough counselling on the risk-benefit profile using prognostic scores [12, 13] or even multigene expression assays [34, 35] should be recommended for informed decision-making. The main challenge remains the selection of low-risk patients, in whom RT provides negligible absolute benefit and can be safely omitted, as well as to identify women at high risk, in order to be able to discuss advantages and disadvantages of a RT boost on an individual basis. Furthermore, patient compliance with follow-up care and the need for regular mammography surveillance play an important role in clinical decision making outside of randomised trials.

## Conclusion

In conclusion, the present study provides insights regarding the adoption of postoperative RT following BCS for DCIS in a large cohort, reflecting “real-life” clinical practice in this setting. Postoperative RT was found to be associated with a reduced risk of ipsilateral recurrence and no survival benefit compared to observation alone.

## Abbreviations

BCS: Breast-conserving therapy
CI: Confidence interval
DCIS: Ductal carcinoma in situ
IBR: In-breast recurrence
OR: Odds ratio
OS: Overall survival
RT: Radiotherapy
